# Dissecting the functions and regulatory mechanisms of disulfidoptosis-related RPN1 in pan-cancer: modulation of immune microenvironment and cellular senescence

**DOI:** 10.3389/fimmu.2024.1512445

**Published:** 2024-12-19

**Authors:** Lexin Qin, Tingting Liang, Hailong Zhang, Xian Gong, Meidan Wei, Xiangrong Song, Yaoyu Hu, Xinyu Zhu, Wentao Hu, Jianxiang Li, Jin Wang

**Affiliations:** School of Public Health, Suzhou Medicine College of Soochow University, Jiangsu, Suzhou, China

**Keywords:** RPN1, disulfidoptosis, endoplasmic reticulum stress, cellular senescence, immune microenvironment, SP1

## Abstract

**Introduction:**

Cancer’s inherent heterogeneity, marked by diverse genetic and molecular alterations, presents significant challenges for developing effective treatments. One such alteration is the regulation of disulfidoptosis, a recently discovered programmed cell death pathway. RPN1, a key regulator associated with disulfidoptosis, may influence various aspects of tumor biology, including immune evasion and cellular senescence. This study aims to dissect the role of RPN1 in pan-cancer and its potential as a therapeutic target.

**Methods:**

We employed a pan-cancer analysis to explore RPN1 expression and its association with clinical outcomes across multiple tumor types. Immune cell infiltration and expression of immune checkpoint genes were analyzed in relation to RPN1. Additionally, cellular senescence markers were assessed in RPN1 knockdown tumor cells. Gene regulatory mechanisms were studied through gene copy number variations, DNA methylation analysis, and transcriptional regulation by SP1.

**Results:**

RPN1 is overexpressed in a wide range of tumor types and correlates with poor clinical outcomes, including overall survival, disease-specific survival, and progression-free intervals. Our analysis shows that RPN1 is involved in immune evasion, correlating with the presence of myeloid dendritic cells, macrophages, and tumor-associated fibroblasts, and influencing T-cell activity. RPN1 knockdown led to reduced tumor cell proliferation and induced cellular senescence, marked by increased senescence-associated biomarkers and β-galactosidase activity. RPN1 expression was found to be regulated by gene copy number variations, reduced DNA methylation, and transcriptional control via SP1.

**Discussion:**

These findings highlight RPN1 as a key pan-cancer regulator, influencing immune microenvironment interactions and cellular senescence. The regulation of disulfidoptosis by RPN1 presents a promising avenue for therapeutic intervention. Targeting RPN1 could enhance immunotherapy efficacy and help mitigate tumor progression, offering a potential strategy for cancer treatment.

## 1 Introduction

In the latest advancements in cell death research, a novel form of programmed cell death known as disulfidptosis has emerged as a significant area of study (1, 2). Dr. Gan from the Cancer Center has detailed this new cell death mechanism, characterized by its unique sensitivity to disulfide stress. Under glucose deprivation, cells with high expression of SLC7A11 exhibit rapid depletion of NADPH, leading to abnormal accumulation of disulfides such as cystine (1). The research emphasizes the intricate relationship between this cell death process and the actin cytoskeleton, with significant changes observed in actin filaments during disulfidptosis (2). As a metabolism-related form of cell death, it holds promise for targeting cancer metabolic vulnerabilities by inducing disulfidptosis, thereby providing a novel avenue for cancer metabolic therapy (3). Several studies have explored the multi-omic characteristics and potential functions of disulfidptosis in bladder cancer (4), esophageal cancer (5), and lung cancer (6). Previous research using CRISPR screening identified potential genes involved in disulfidptosis, further screening and validating the key gene NCKAP1, a critical regulator of actin filament aggregation and lamellipodia formation, underscoring the role of the cytoskeleton in this novel cell death pathway (2).

RPN1, a member of the glycosyltransferase family 13, has also been found to be strongly associated with disulfidptosis (2). RPN1 plays a crucial role in glycosylation, enabling interactions with other biomolecules to exert its biological functions (7, 8). Knockdown of RPN1-mediated aberrant protein hypoglycosylation can activate endoplasmic reticulum stress (ERS), thereby inhibiting the proliferation and invasion of breast cancer cells, promoting apoptosis, and suppressing tumor progression (8). Despite the potential significance of RPN1, its comprehensive role across various cancers remains underexplored.

Pan-cancer research, which integrates data across different tumor types, represents a transformative approach to understanding the complex landscape of cancer biology. This broad-spectrum analysis can identify shared molecular pathways and genetic alterations that might be obscured in single-cancer studies (9, 10). By examining these commonalities, pan-cancer research can reveal universal mechanisms of tumorigenesis and treatment resistance, uncovering new intervention targets. This comprehensive approach also aids in discovering biomarkers applicable to multiple cancer types, potentially leading to more effective and widely applicable treatment strategies, thereby promoting the development of more precise and inclusive therapeutic models (9).

This study aims to address this gap by systematically analyzing the expression and biological and clinical significance of RPN1 across various cancer types using bioinformatics approaches. By leveraging large-scale cancer datasets, we will elucidate the differential expression patterns and prognostic value of RPN1, thereby enhancing our understanding of its involvement in tumor biology. Additionally, we will validate these findings through *in vitro* and *in vivo* experiments to elucidate the functional relevance of RPN1 and its potential as a therapeutic target, providing valuable insights into the complex mechanisms of cancer cell death and survival.

## 2 Methods

### 2.1 Data source

The results of whole-genome CRISPR–Cas9 screening in SLC7A11-overexpressing 786-O cells under glucose-replete and -starved conditions were downloaded from previous study (2).

The log2 (TPM + 0.001) transformed normalized gene and transcripts expression profiles, copy number variations on gene expression estimated using the Genomic Identification of Significant Targets in Cancer 2.0 (GISTIC2.0) method, DNA methylation profiles and phenotype data of pan-cancer were download from the University of California, Santa Cruz Genome Browser (UCSC) Xena database (https://xenabrowser.net/). Moreover, the proteomics data of multiple cancer types were obtained from the Clinical Proteomic Tumor Analysis Consortium (CPTAC) database (https://proteomics.cancer.gov/programs/cptac). The ProteoCancer Analysis Suite (PCAS) package was used to analyze the CPTAC datasets (11). Also, several datasets for various cancer types were obtained from GEO datasets (https://www.ncbi.nlm.nih.gov/geo/).

### 2.2 Correlation analysis

To understand the biological and clinical significance of RPN1, correlations were analyzed between the expression of RPN1 and genesets expression, Tumor Mutation Burden (TMB), immune cell infiltration, and Tumor Immune Dysfunction and Exclusion (TIDE) score, as well as drug sensitivity score. Oncogenes were obtained from ONGene (http://www.ongene.bioinfo-minzhao.org) (12) databases. A total of 11 immune checkpoint genes (ICGs) were extracted from previous studies (13). Immune cell infiltration scores came from the TIMER2.0 database (http://timer.cistrome.org/) (14). TIDE scores were predicted using the online TIDE tool (http://tide.dfci.harvard.edu/) (15). Data from the Genomics of Drug Sensitivity in Cancer database (GDSC, https://www.cancerrxgene.org/) was used to assess drug sensitivity using “oncoPredict” (16). Spearman’s method (“psych” package) was used for correlations.

### 2.3 Enrichment analysis

Gene set enrichment analysis (GSEA) was performed using the “ClusterProfiler” R package to explore gene set enrichment based on correlation analysis. Specifically, two gene set collections were employed: c2.cp.kegg.v2023.2.entrez.gmt for signaling pathway analysis and c5.go.bp.v2023.2.entrez.gmt for biological processes associated with RPN1. This analysis spanned multiple cancer types to identify consistently enriched pathways and biological processes. Following GSEA for each cancer type, commonly enriched gene sets were extracted and visualized to underscore their significance.

### 2.4 RPN1 shRNA plasmid construction

RPN1 shRNA sequences were designed using the BLOCK-iT™ RNAi Designer tool (Thermo Fisher Scientific, URL: https://rnaidesigner.thermofisher.com/rnaiexpress). The annealed shRNA double-stranded fragments were cloned into the pGreen vector. After assessing the knockdown efficiency of multiple candidate shRNAs, two shRNAs targeting RPN1 were selected for subsequent experiments. Additionally, a scrambled non-specific control shRNA (shNC) was cloned into the same vector and used as a negative control.

### 2.5 Cell culture and transfection

Human cancer cell lines, including A549 and H1299 (lung cancer), T24 (bladder cancer), SW480 (colon cancer), MDA-MB-231 (breast cancer), MGC-803 (gastric cancer), and U87 (glioblastoma), were acquired from the American Type Culture Collection (ATCC). Cells were cultured in DMEM supplemented with 10% FBS at 37°C in a 5% CO_2_ atmosphere. After a 24-hour incubation period, lung cancer cells were transfected with 2.5 μg of shRNA using Lipofectamine 6000 reagent (Beyotime, China) according to the manufacturer’s instructions.

### 2.6 Cell proliferation assay

For the EdU assay, cells were treated with 10 μM EdU for 2 hours, fixed with 4% paraformaldehyde, and permeabilized with 0.3% Triton X-100 in PBS. The Click reaction solution from Beyotime Institute of Biotechnology (China) was used for subsequent processing. Following a 24-hour incubation, cell images were captured using an inverted fluorescent microscope and analyzed using NIH ImageJ software (Version 1.8.0).

For the CCK-8 assay, cells were incubated with the CCK-8 working solution for 1-4 hours at 37°C. Absorbance was measured at 450 nm using a microplate reader, and the absorbance values were used to calculate cell viability percentages relative to control samples.

### 2.7 qPCR assay

Total RNA was extracted from cultured cells using a commercially available RNA isolation kit, following the manufacturer’s protocol. RNA concentration and purity were assessed using a spectrophotometer (NanoDrop 2000, Thermo Fisher Scientific, USA). cDNA synthesis was performed using a reverse transcription kit (cDNA Reverse Transcription Kit, Thermo Fisher Scientific, USA) according to the manufacturer’s instructions. For qPCR, we used specific primers for CDKN1A (Forward: TGTCCGTCAGAACCCATGC, Reverse: AAAGTCGAAGTTCCATCGCTC) and CDKN2A (Forward: GGGTTTTCGTGGTTCACATCC, Reverse: CTAGACGCTGGCTCCTCAGTA), with a reference gene, ACTB (Forward: CATGTACGTTGCTATCCAGGC, Reverse: CTCCTTAATGTCACGCACGAT). The PCR reactions were performed on a real-time PCR system (ABI Q6, Applied Biosystems, USA), using SYBR Green assay. The thermal cycling conditions were as follows: 95°C for 10 min, followed by 40 cycles of 95°C for 15 sec, 60°C for 1 min. Relative expression levels were calculated using the comparative Ct method (ΔΔCt).

### 2.8 Detection of cellular senescence

Senescent cells were identified using a fluorescein-based probe targeting β-galactosidase activity. The probe, containing two galactoside moieties, is cleaved by β-galactosidase within lysosomes under acidic conditions, emitting a fluorescent signal with absorption/emission maxima of 490/514 nm. Post-incubation with the probe, cells were fixed and nuclei counterstained with DAPI. Fluorescent signals were visualized using a fluorescence microscope equipped with standard filter sets.

### 2.9 Western blot analysis

Protein expression levels of Ki67, P21, and P16 were assessed by Western blotting, with ACTB serving as the internal control. Protein samples were resolved by SDS-PAGE, transferred to PVDF membranes (Millipore, USA), and incubated with primary antibodies: Ki67 (Catalog No. 27309-1-AP, 1:1000 dilution), P21 (Catalog No. 10355-1-AP, 1:1000 dilution), P16 (Catalog No. 10883-1-AP, 1:1000 dilution), sourced from Proteintech (Rosemont, IL, USA). Membranes were then incubated with HRP-conjugated secondary antibodies (Catalog No. SA00001-2/1, 1:5000 dilution, Rosemont, IL, USA) at a 1:5000 dilution for 1 hour at room temperature. Bands were visualized using an enhanced chemiluminescence kit (Beyotime, China). The band intensity was quantified using ImageJ software (NIH, Bethesda, MD, USA), normalized against ACTB levels for accurate comparison across samples.

### 2.10 ELISA assay

ELISA was conducted to measure IL6 and IL8 concentrations in the culture supernatants of RPN1 knockdown cells. ELISA kits for IL6 and IL8 were purchased from R&D Systems (Minneapolis, MN, USA). Results were presented as mean values for shRPN1 and shNC groups, with standard curves generated for each cytokine to ensure accurate quantification. Data analysis was performed using GraphPad Prism software (GraphPad Software, San Diego, CA, USA) to compare cytokine levels between the RPN1 knockdown and control conditions.

### 2.11 Nude mouse tumorigenesis assay

All animal experiments adhered to institutional animal ethics guidelines and were conducted following the ethical standards approved by the Laboratory Animal Ethics Committee of the Experiment Animal Center of Soochow University (approval no. 202401A052). Six nude mice per group were used: one group received subcutaneous injections of H1299 cells transfected with shNC, while the other group received shRPN1-transfected H1299 cells. Tumor growth was monitored weekly by recording body weight and tumor volume.

At the endpoint of the study, all animals were euthanized humanely under deep anesthesia to minimize suffering. Anesthesia was achieved using an intraperitoneal injection of pentobarbital sodium at a dose of 50 mg/kg. Following confirmation of deep anesthesia, as indicated by the loss of the righting reflex and absence of response to a toe pinch, euthanasia was performed by cervical dislocation. After euthanasia, tumors were promptly excised, weighed, and analyzed. Tumor volumes were calculated using the formula V = (length × width²)/2. Body weight and tumor size data were used to evaluate the impact of RPN1 knockdown on tumor growth.

### 2.12 Immunohistochemistry

Tumor tissues were processed into paraffin-embedded sections and subjected to standard immunohistochemistry protocols. Sections were incubated with primary antibodies against RPN1(Catalog No. 12894-1-AP, 1:200 dilution), SLC7A11 (Catalog No. 26864-1-AP, 1:200 dilution), P16 (Catalog No. 10883-1-AP, 1:200 dilution), Ki67 (Catalog No. 27309-1-AP, 1:200 dilution), and CD31 (Catalog No. 11265-1-AP, 1:200 dilution), all from Proteintech (Rosemont, IL, USA). Quantitative analysis was conducted using ImageJ software (NIH, Bethesda, MD, USA) to determine the Area of Differential Staining (AOD) for RPN1, SLC7A11, and P16, the proliferation index for Ki67-positive cells, and the blood vessel areas for CD31-positive cells.

### 2.13 Copy number analysis

Copy number analysis was performed using qPCR with an absolute quantification method. DNA samples were extracted from cells and subjected to qPCR using specific primers targeting RPN1 (Forward: GGCCAAGATTTCAGTCATTGTGG, Reverse: CTTCGTTGGATAGGGAGAGTAGA). Standard curves from known copy number standards quantified absolute copy numbers per cell. Results were expressed as copy numbers per cell, with a copy number of 2 serving as the baseline control. All qPCR reactions were performed in triplicate, and data were analyzed using the relative quantification method.

### 2.14 Methylation-specific PCR

Genomic DNA was extracted using a DNA Purification Kit, and 1 μg of DNA was modified with sodium bisulfite using the Epitect Bisulfite kit (Qiagen, Inc.) according to the manufacturer’s specifications. MSP was conducted in a 20 μl reaction volume using AmpliTaq Gold (Applied Biosystems; Thermo Fisher Scientific, Inc.). MSP products were separated on 2% agarose gels containing GelRed^®^ Nucleic Acid Gel Stain. Successful bisulfite modification was confirmed by amplifying the GAPDH promoter region with modified DNA samples. Primers used were: MF: 5’-AATTGTTATGTTGTTTATTTTTCGA-3’, MR: 5’-ACTACGACCTACCTTTATATACGAA-3’, UF: 5’-AATTGTTATGTTGTTTATTTTTTGA-3’, UR: 5’-AACTACAACCTACCTTTATATACAAA-3’.

### 2.15 Transcription factor prediction

Using TFTF R package to predict upstream transcription factors regulating RPN1 (17), a total of 7 transcription factor target gene prediction datasets were included, including KnockTF (18), hTFTarget (19), ChIP_Atlas (20), GTRD (21), and ENCODE (22), as well as prediction results calculated using FIMO algorithm based on core transcription factors from JASPAR database (23, 24). Further calculate the expression correlation between RPN1 and SP1 based on the pan organizational dataset of TCGA and GETx databases.

### 2.16 Dual-luciferase reporter assay

To investigate the regulatory effect of the transcription factor SP1 on the target gene RPN1, a dual-luciferase reporter assay was performed. The complete promoter sequence of RPN1 was amplified and cloned into a firefly luciferase reporter vector (pGL3-basic, Promega, Madison, WI, USA). The control vector containing a Renilla luciferase gene was used for normalization of transfection efficiency (pRL-TK, Promega, Madison, WI, USA).

Cells were co-transfected with the RPN1 promoter-firefly luciferase construct and the Renilla luciferase control vector, along with or without a SP1 expression vector. After 48 hours, the cells were lysed, and luciferase activities were measured using a Dual-Luciferase Reporter Assay System (Promega, Madison, WI, USA) according to the manufacturer’s instructions. The firefly luciferase activity was normalized to the Renilla luciferase activity to determine the relative promoter activity. The fold change in luciferase activity in the presence of SP1 was compared to the control to assess the regulatory impact of SP1 on the RPN1 promoter.

### 2.17 Statistical analysis

Spearman method is used for correlation analysis. The results from *in vitro* experiments are presented as means ± standard deviation, and were analyzed using SPSS 22.0 software (IBM Corp.). Differences between groups were analyzed using Student’s t-test or one-way ANOVA as appropriate. P < 0.05 was considered to indicate a statistically significant difference.

## 3 Results

### 3.1 RPN1 promotes glucose deprivation-induced cell death

**Figure 1:**
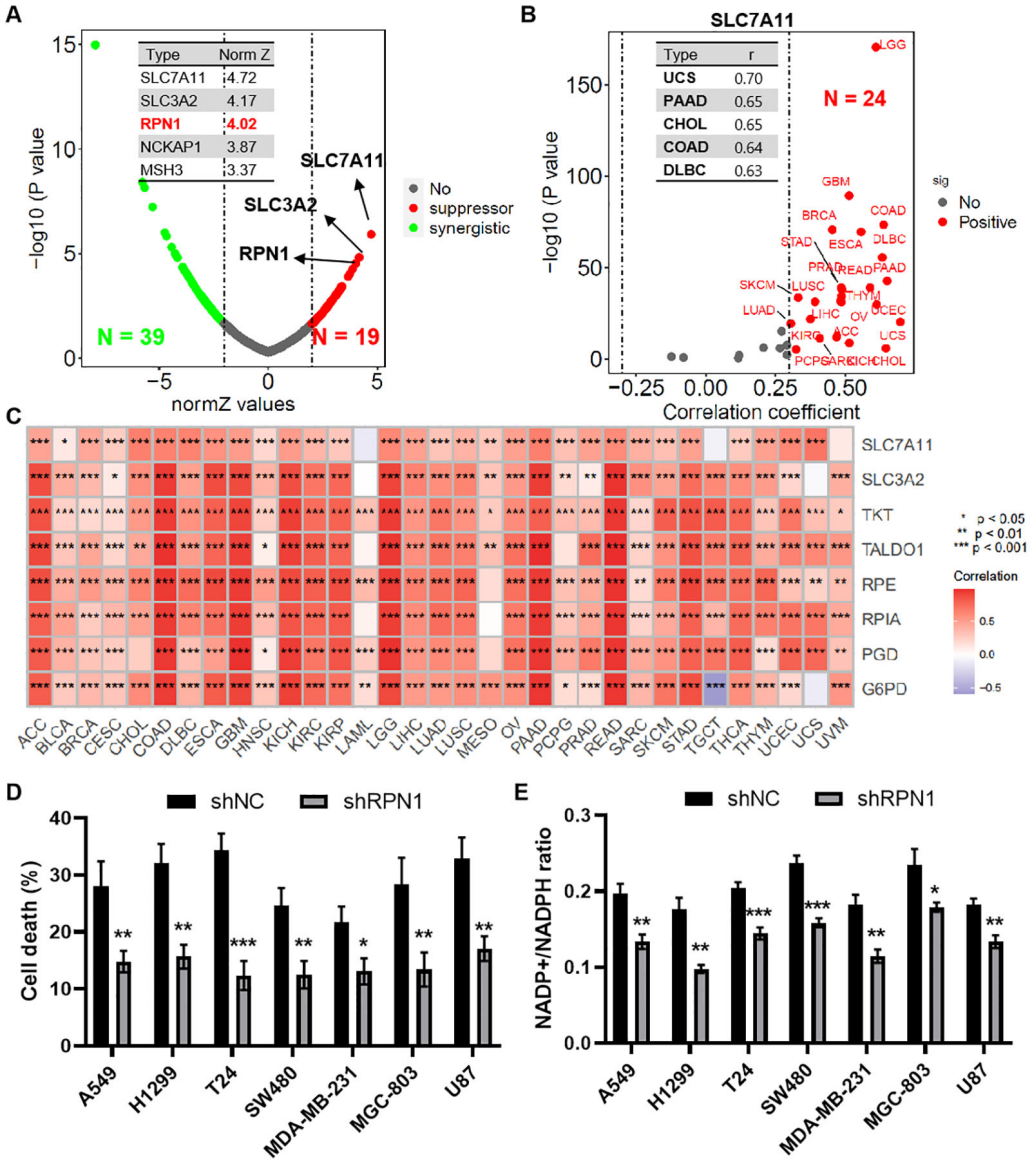
RPN1 promotes glucose deprivation-induced cell death. **(A)** Scatter plot showing results of the whole-genome CRISPR–Cas9 screening. **(B)** Scatter plot showing correlation analysis between SLC7A11 and RPN1 expression across multiple tumor types in the TCGA database. **(C)** Heat map showing correlation analysis between RPN1 expression and pentose phosphate pathway (PPP)-related genes in multiple tumor types. * P < 0.05, ** P < 0.01,*** P < 0.001. **(D)** CCK-8 assay showing the effect of RPN1 knockdown on cell death after 8 hours of glucose deprivation. **(E)** Effect of RPN1 knockdown on the NADP+/NADPH ratio after glucose deprivation. Compare with shNC, * P < 0.05, ** P < 0.01, *** P < 0.001. TCGA, The Cancer Genome Atlas. NADPH, Nicotinamide adenine dinucleotide phosphate.

Based on results from a whole-genome CRISPR-Cas9 screening of SLC7A11-overexpressing 786-O cells under glucose-sufficient and glucose-starved conditions, RPN1 emerged as the third-ranked suppressor hit gene (Norm Z = 4.02, **Figure 1A**). Correlation analysis from the TCGA database indicated a significant positive correlation between RPN1 and SLC7A11 expression in 24 types of tumors (r > 0.3, P < 0.05, **Figure 1B**). The top five tumors by this correlation were UCS (P = 0.70), PAAD (P = 0.65), CHOL (P = 0.65), COAD (P = 0.64), and DLBC (P = 0.63). Further correlation analysis demonstrated a significant positive correlation between RPN1 expression and key genes of the pentose phosphate pathway in the majority of tumors (P < 0.05, **Figure 1C**). Notably, SLC3A2 expression showed a significant positive correlation with RPN1 in 27 types of tumors (r > 0.3, P < 0.05, **Supplementary Figure S1A**), and TKT expression showed a significant positive correlation with RPN1 in 26 types of tumors (r > 0.3, P < 0.05, **Supplementary Figure S1B**). To validate the role of RPN1 in promoting disulfide-induced cell death, RPN1 was knocked down in eight types of tumor cells (**Supplementary Figure S2**), which were then cultured in glucose-deprived medium. Knockdown of RPN1 significantly rescued the increase in cell death induced by 8 hours of glucose deprivation (**Figure 1D**) and the upregulation of the NADP+/NADPH ratio (**Figure 1E**).

### 3.2 RPN1 is broadly upregulated and a prognostic risk factor in pan-cancer

**Figure 2:**
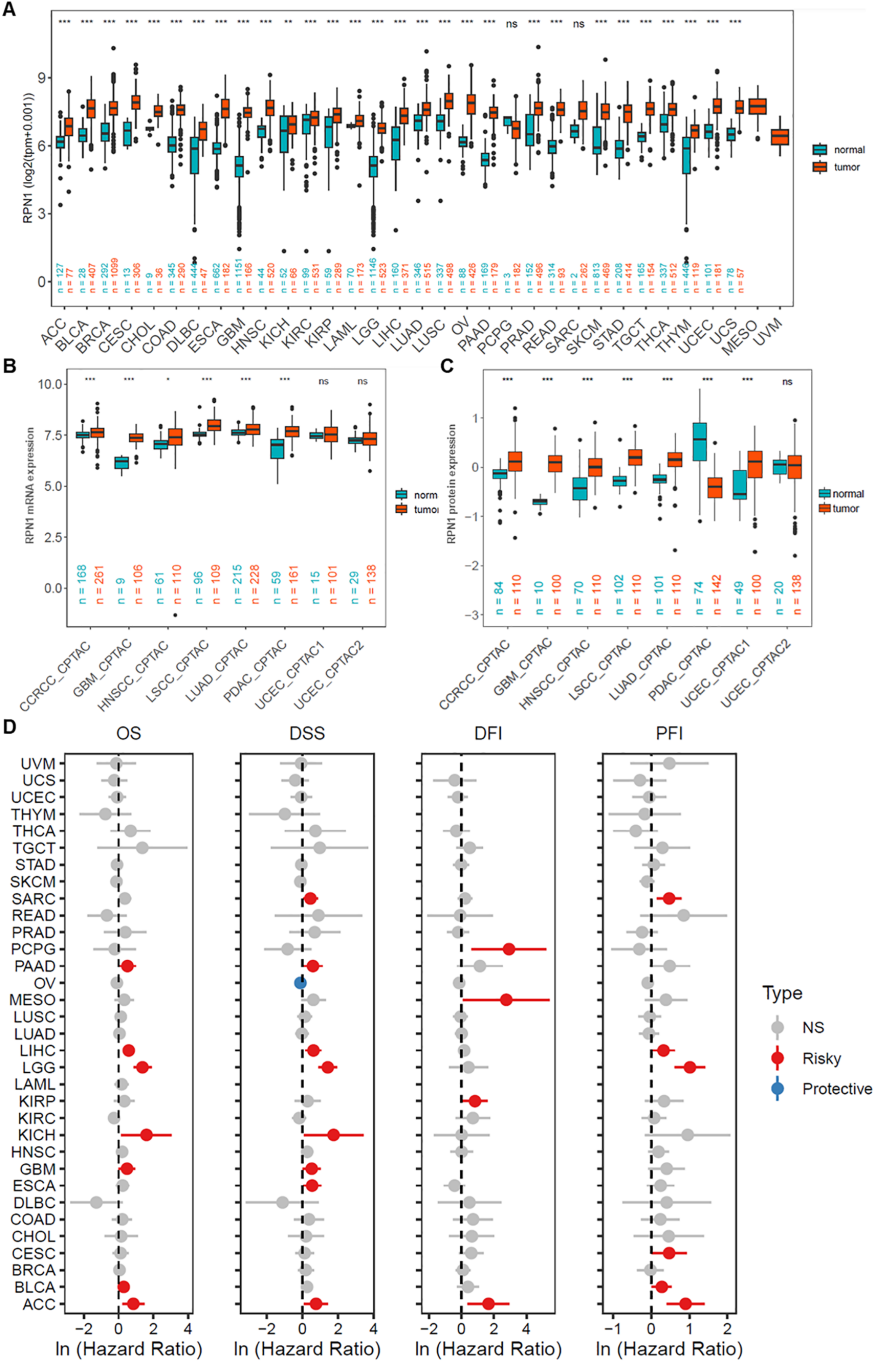
RPN1 is broadly upregulated and a prognostic risk factor in pan-cancer. **(A)** Analysis of RPN1 transcript levels across multiple cancers from the combined TCGA and GTEx dataset. **(B)** Analysis of RPN1 transcript levels across multiple cancers from the CPTAC database. **(C)** Analysis of RPN1 protein levels across multiple cancers from the CPTAC database. **(D)** Analysis of the correlation between RPN1 expression and OS, DSS, DFI, and PFI in patients with various cancers from the TCGA database. TCGA, The Cancer Genome Atlas. CPTAC, Clinical Proteomic Tumor Analysis Consortium. GTEx, Genotype-Tissue Expression. OS, Overall Survival. DSS, Disease-Specific Survival. DFI, Disease-Free Interval. PFI, Progression-Free Interval. ns/NS, not significant, * P < 0.05, ** P < 0.01, *** P < 0.001.

Based on the pan-cancer dataset combining TCGA and GTEx databases, RPN1 was found to be significantly upregulated in the majority of tumor types (P < 0.05, **Figure 2A**). Furthermore, transcriptomic data from the CPTAC cancer dataset also showed significant upregulation of RPN1 in several tumors (P < 0.05, **Figure 2B**). Additionally, high-throughput transcriptomic data for various cancer types obtained from the GEO database indicated that RPN1 is significantly upregulated in multiple tumors (P < 0.05, **Supplementary Figure S3**). Moreover, paired t-test analysis revealed that RPN1 is significantly upregulated in paired tumor samples across several cancer types (P < 0.05, **Supplementary Figure S4**). According to the Ensembl database, RPN1 has eight transcripts, including two protein-coding transcripts (**Supplementary Figure S5A**), all of which are significantly upregulated in multiple tumors based on TCGA data (**Supplementary Figure S5B**). From the CPTAC database, we observed that RPN1 protein expression is significantly higher in various cancers compared to normal tissues (P < 0.05, **Figure 2C**). Importantly, further COX analysis indicated that RPN1 is correlated with OS, DSS, DFI, and PFI in various cancers, serving as a risk factor (HR > 1, P < 0.05, **Figure 2D**). Using the KMplotter online tool, high RPN1 expression was associated with poorer prognosis in patients with lung, colon, and breast cancers (HR > 1, P < 0.05, **Supplementary Figures S6A-C**).

### 3.3 RPN1 is associated with tumor biological processes and signaling pathways

**Figure 3:**
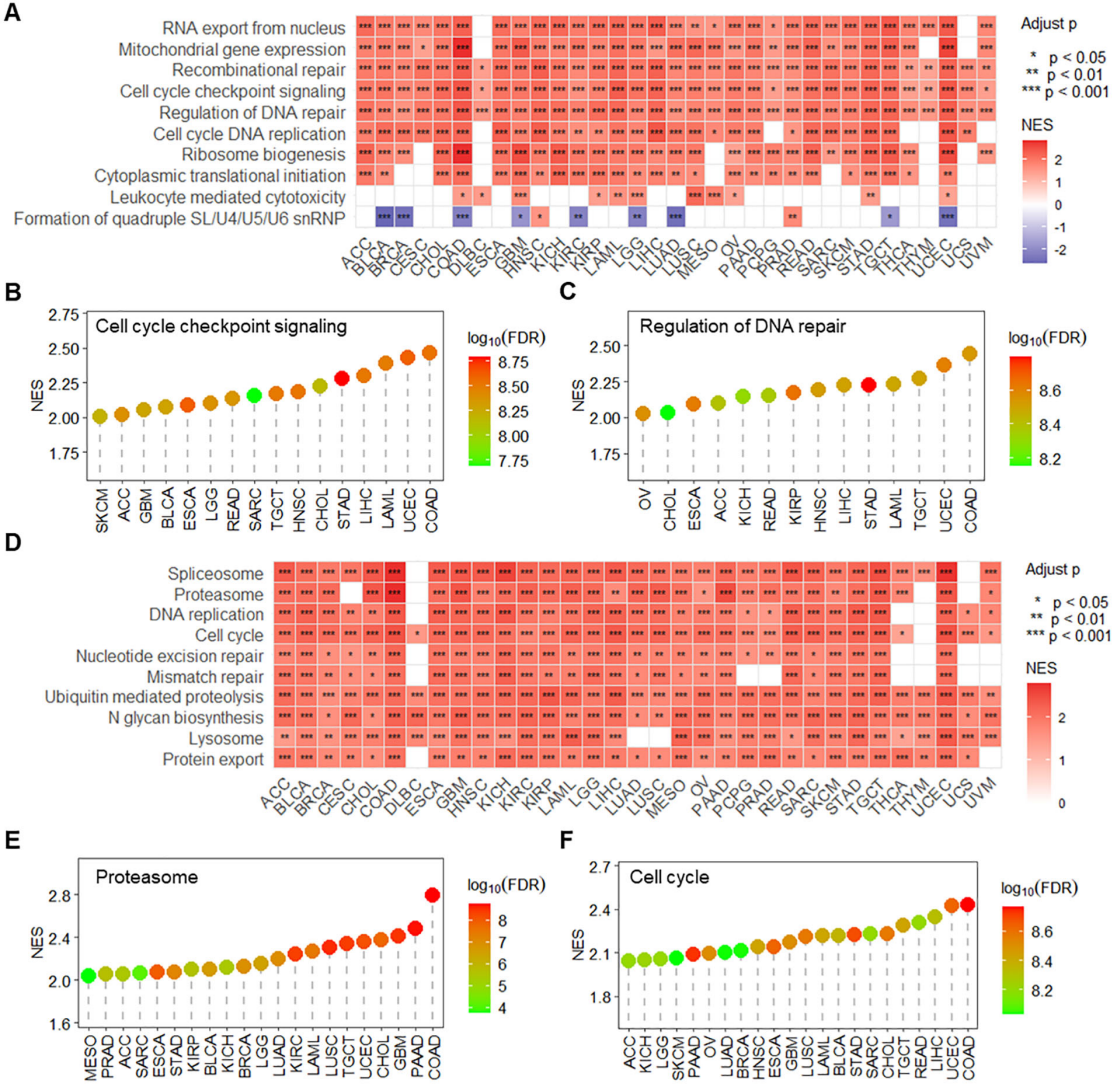
RPN1 is associated with tumor-related signaling pathways and biological functions. **(A)** Heatmap displaying the GSEA enrichment analysis results of RPN1 expression-associated genes in biological processes across pan-cancer samples. lollipop charts showing the enrichment results of RPN1 expression-associated genes in the “Cell cycle checkpoint signaling” term **(B)** and “Regulation of DNA repair” term **(C)** across various cancers. **(D)** Heatmap displaying the GSEA enrichment analysis results of RPN1 expression-associated genes in KEGG pathways across pan-cancer samples. Lollipop charts showing the enrichment results of RPN1 expression-associated genes in the “Proteasome” term **(E)** and “Cell cycle” term **(F)** across various cancers. GSEA, Gene Set Enrichment Analysis. * P < 0.05,** P < 0.01,*** P < 0.001.

Based on GSEA enrichment analysis, RPN1 was found to be significantly enriched in multiple key biological processes across various tumors (**Figure 3A**), including mitochondrial gene expression, recombinational repair, leukocyte-mediated cytotoxicity, cell cycle checkpoint signaling (**Figure 3B**), and regulation of DNA repair (**Figure 3C**). Regarding KEGG signaling pathways, enrichment analysis revealed that RPN1 is significantly associated with several critical biological functions (**Figure 3D**), primarily including proteasome (**Figure 3E**), cell cycle (**Figure 3F**), spliceosome, DNA replication, nucleotide excision repair, mismatch repair, lysosome, and protein export. To further evaluate the significance of RPN1 in cancer progression, we analyzed the correlation between RPN1 expression and oncogene expression across pan-cancer samples. The results indicated that RPN1 is significantly positively correlated with oncogene expression in most tumors (P < 0.05, **Supplementary Figure S6A**). Specifically, correlation analysis showed that the oncogenes HSPA5 and TFG are significantly positively correlated with RPN1 expression in nearly all cancers (P < 0.05, r > 0.2, **Supplementary Figures S7B, C**).

### 3.4 RPN1 contributes to endoplasmic reticulum stress

**Figure 4:**
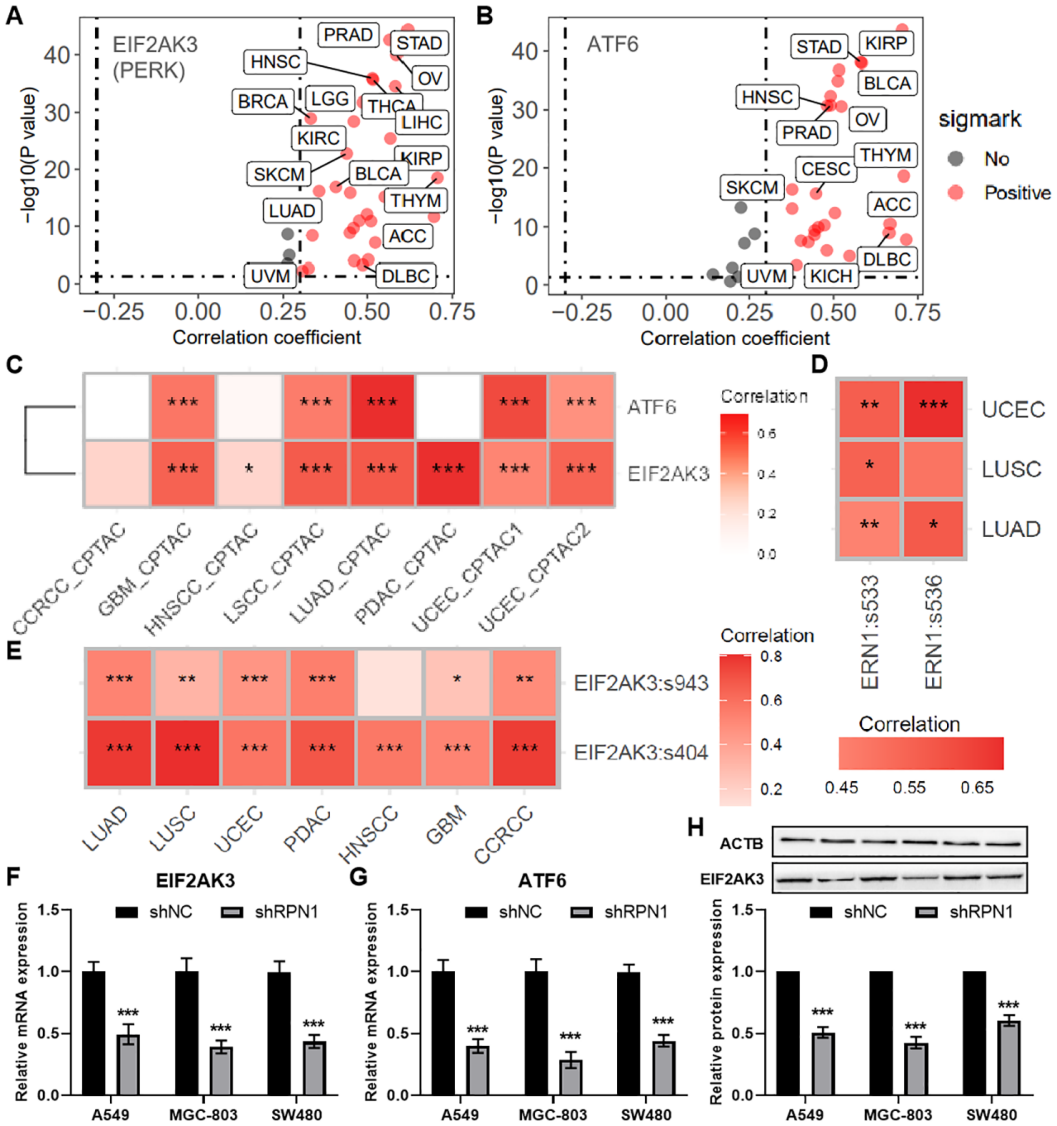
RPN1 Contributes to Endoplasmic Reticulum Stress. Scatter plots showing the correlation analysis results between the mRNA expression of RPN1 and PERK **(A)** and ATF6 **(B)** based on the pancancer data of TCGA database. **(C)** Heatmap showing the correlation between the protein expression of RPN1 and PERK and ATF6 based on PCAS package. Heatmap showing the correlation between the protein expression of RPN1 and the phosphorylation levels of ERN1 **(D)** and PERK **(E)** based on PCAS package. * P < 0.05,** P < 0.01,*** P < 0.001. qPCR analysis showing changes in PERK **(F)** and ATF6 **(G)** expression in the three cell lines following RPN1 knockdown. Western blot analysis showing representative images and statistical results of PERK protein expression changes in the three cell lines after RPN1 knockdown. TCGA, The Cancer Genome Atlas. PCAS, ProteoCancer Analysis Suite. Compare with shNC, * P < 0.05, ** P < 0.01, *** P < 0.001.

Based on enrichment analysis, RPN1 was found to be associated with the proteasome, prompting us to further investigate its relationship with endoplasmic reticulum (ER) stress. Correlation analysis using transcriptomic data from the TCGA database revealed that RPN1 expression positively correlates with the ER stress-related genes PERK (EIF2AK3, **Figure 4A**) and ATF6 (**Figure 4B**) in multiple cancer types. Furthermore, using the PCAS package, we observed that RPN1 protein expression also positively correlates with the levels of PERK and ATF6 in various tumors (**Figure 4C**). Phosphorylation data from the PCAS package analysis revealed that RPN1 levels significantly positively correlate with the phosphorylation of IRE1α (ERN1, **Figure 4D**) and PERK (**Figure 4E**). Moreover, in three cancer cell lines (A549, MGC-803, and SW480), we analyzed the expression changes of PERK and ATF6 following RPN1 knockdown. The results showed that both PERK and ATF6 were significantly downregulated (**Figures 4F, G**). At the protein level, PERK expression was significantly decreased following RPN1 knockdown (**Figure 4H**).

### 3.5 Knockdown of RPN1 inhibits tumor cell proliferation and promotes cellular senescence

**Figure 5:**
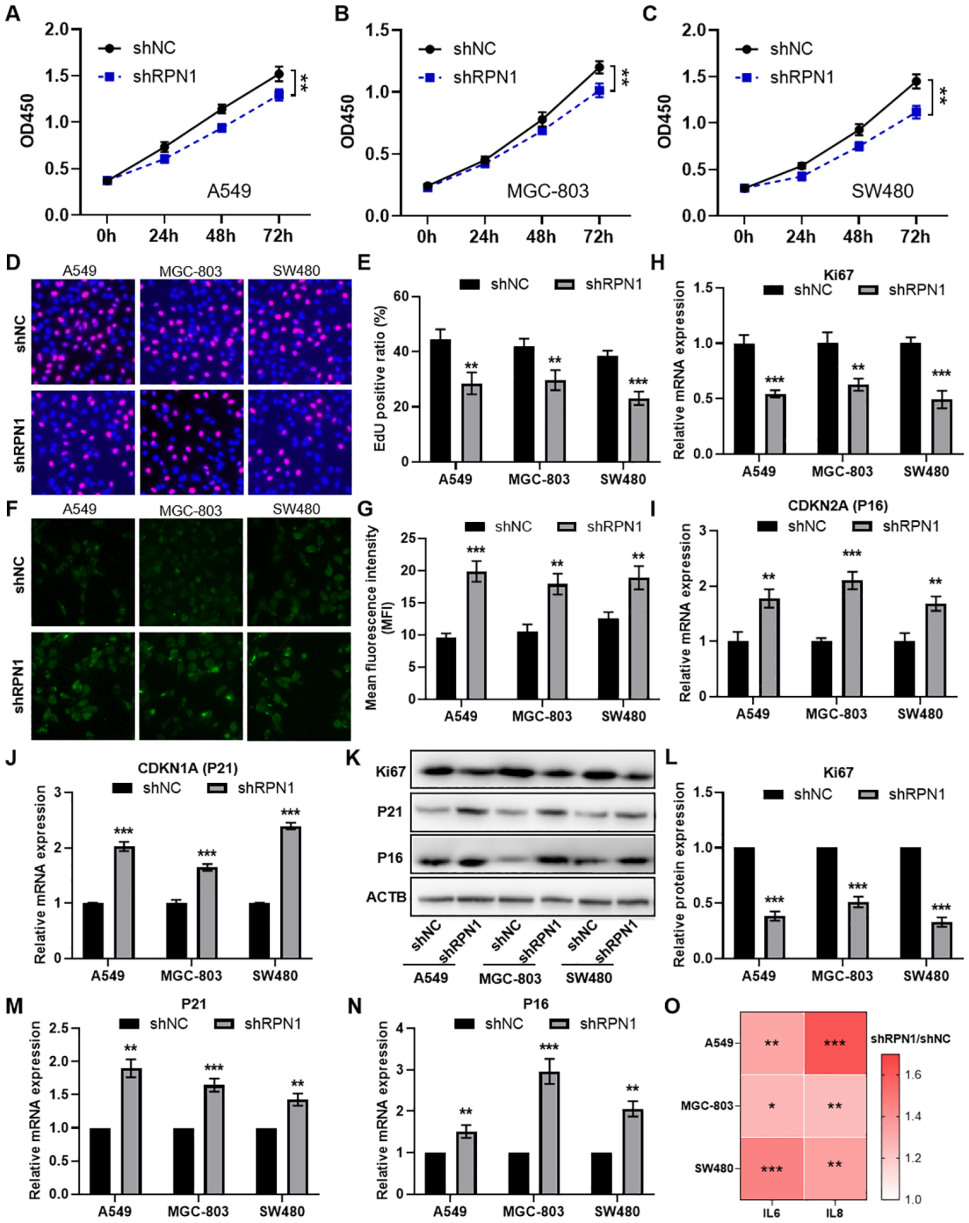
Knockdown of RPN1 inhibits cell cycle and promotes cellular senescence. CCK-8 assay analyzing the proliferation changes in A549 **(A)**, MGC-803 **(B)**, and SW480 **(C)** cells following RPN1 knockdown. EdU assay showing representative images **(D)** and statistical results **(E)** of proliferation changes in the three cell lines after RPN1 knockdown. immunofluorescence staining for β-galactosidase activity showing representative images **(F)** and statistical results **(G)** of senescence in the three cell lines after RPN1 knockdown. qPCR analysis showing changes in Ki67 **(H)**, CDKN2A **(I)**, and CDKN1A **(J)** expression in the three cell lines following RPN1 knockdown. Western blot analysis showing representative images **(K)** and statistical results **(L-N)** of Ki67, P16, and P21 protein expression changes in the three cell lines after RPN1 knockdown. Compare with shNC, ** P < 0.01, *** P < 0.001. **(O)** ELISA detecting changes in IL6 and IL8 concentrations in the culture supernatants of the three cell lines following RPN1 knockdown. * P < 0.05, ** P < 0.01, *** P < 0.001.

To further investigate the regulatory effects of RPN1 on cell proliferation and senescence, we constructed RPN1 knockdown plasmids and conducted *in vitro* experiments. CCK-8 assays demonstrated that knockdown of RPN1 significantly reduced the proliferative capacity of lung cancer cells A549 (**Figure 5A**), gastric cancer cells MGC-803 (**Figure 5B**), and colon cancer cells SW480 (**Figure 5C**). EdU incorporation assays visually confirmed the reduced proliferation in these cell lines, as shown by representative images (**Figure 5D**) and quantification results (**Figure 5E**). Immunofluorescence staining for β-galactosidase activity indicated increased cellular senescence following RPN1 knockdown (**Figures 5F, G**). Further correlation analysis indicated that RPN1 expression is significantly positively correlated with cell cycle-related gene expression in LUAD, STAD, and COAD (**Supplementary Figures S8A-C**). qPCR analysis demonstrated that knockdown of RPN1 led to a significant downregulation of several key cell cycle regulatory genes (**Supplementary Figure S8D**). In addition, Ki67 mRNA levels were downregulated in RPN1-depleted cells (**Figure 5H**), whereas CDKN2A (**Figure 5I**) and CDKN1A (**Figure 5J**) mRNA levels were upregulated. These findings were corroborated at the protein level by Western blot analysis, which showed decreased Ki67 and increased expression of P16 (product of CDKN2A) and P21 (product of CDKN1A) (**Figures 5K-N**). Additionally, ELISA detected elevated concentrations of IL6 and IL8 in the culture supernatants of RPN1-knockdown cells, indicating enhanced secretion of senescence-associated inflammatory cytokines (**Figure 5O**).

### 3.6 Knockdown of RPN1 inhibits tumor formation in nude mice

**Figure 6:**
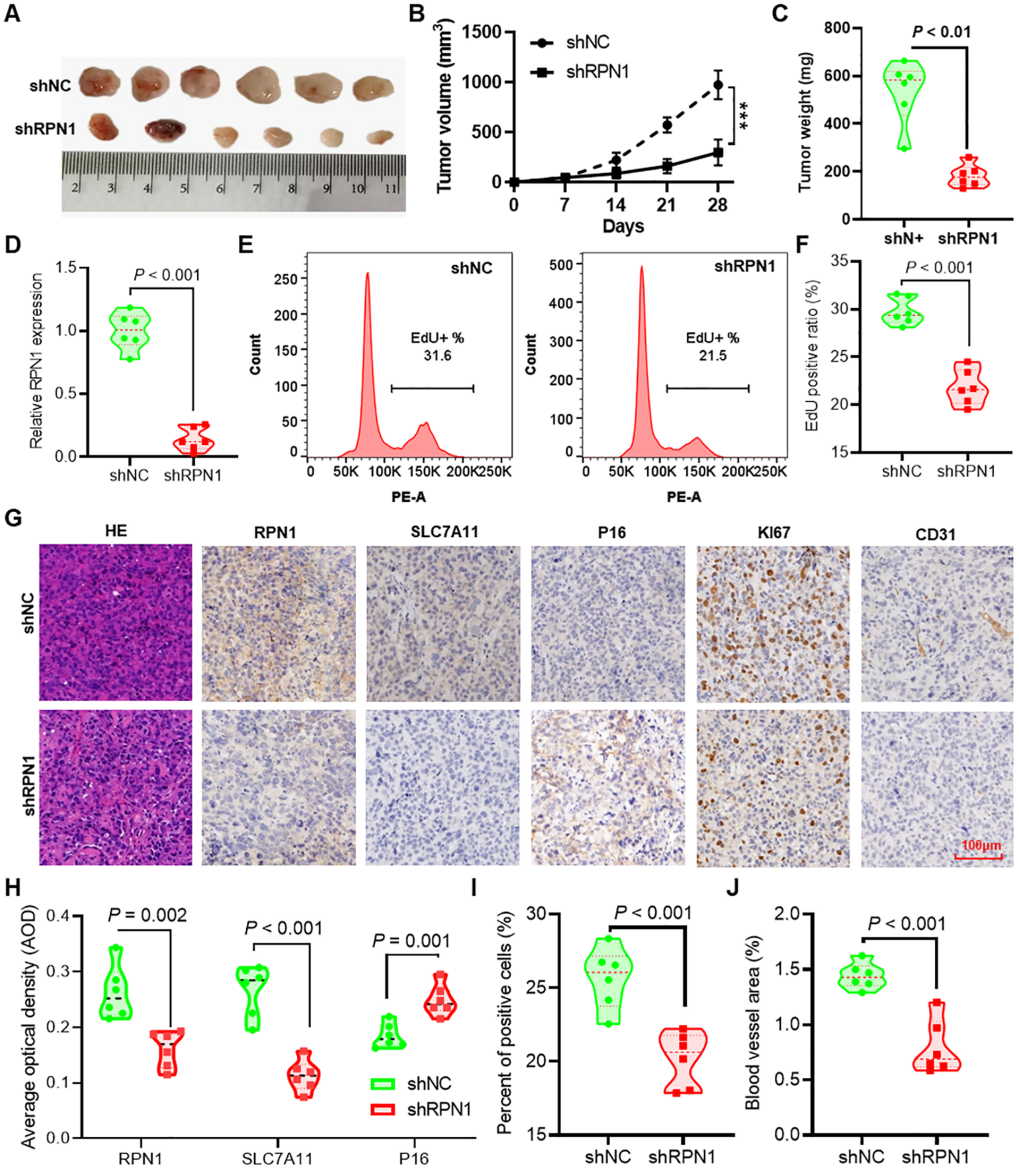
Knockdown of RPN1 inhibits tumor formation in nude mice. **(A)** Images of subcutaneous tumors formed by H1299 lung cancer cells stably transfected with shNC and shRPN1 in nude mice. **(B)** Growth curves of subcutaneous tumors in nude mice. *** P < 0.001. **(C)** Differences in tumor weights at the experimental endpoint between the two groups of mice. **(D)** qPCR detection of differences in RPN1 expression between tumors from the two groups of mice. Representative images **(E)** and statistical results **(F)** of EdU proliferation assays on dissociated tumor tissues from the two groups of mice. **(G)** Immunohistochemical staining analysis of RPN1, SLC7A11, P16, Ki67, and CD31 expression differences in tumor tissues from the two groups of mice. **(H)** AOD statistical results of immunohistochemical images for RPN1, SLC7A11, and P16. **(I)** Statistical results of the proportion of Ki67-positive cells in immunohistochemical staining images. **(J)** Statistical results of the vascular area in CD31 immunohistochemical staining images. AOD, average optical density.

We demonstrated the effects of RPN1 knockdown on subcutaneous tumor formation using shNC and shRPN1 stably transfected H1299 cancer cells in nude mice (**Figure 6A**). Tumor growth curves indicated that the shRPN1 group exhibited significantly reduced tumor growth compared to the shNC group (**Figure 6B**). At the end of the experiment, the tumor weights in the shRPN1 group were markedly lower (**Figure 6C**). qPCR analysis confirmed reduced RPN1 expression in tumors from the shRPN1 group (**Figure 6D**). Dissociation of tumor tissues for EdU proliferation assays revealed inhibited proliferation of tumor cells in the shRPN1 group (**Figures 6E, F**). Immunohistochemical staining indicated significantly decreased expression of RPN1 and SLC7A11, and increased expression of P16 in the shRPN1 group (**Figures 6G, H**). Furthermore, quantitative analysis showed a reduced proportion of Ki67-positive cells in the tumors from the shRPN1 group (**Figure 6I**), along with a decreased area of intratumoral vasculature (**Figure 6J**).

### 3.7 Regulation of RPN1 gene expression by copy number alterations and DNA methylation

**Figure 7:**
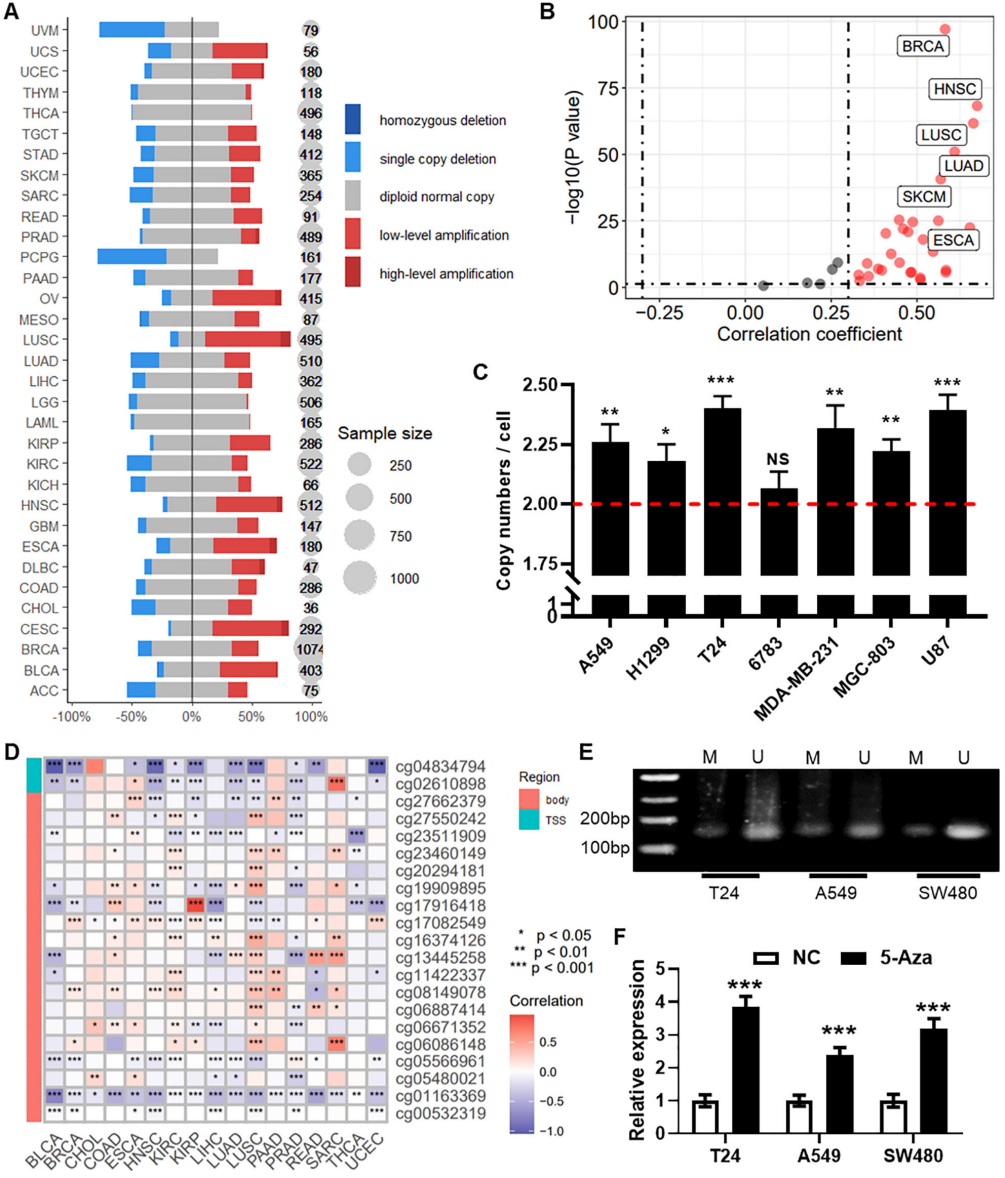
Regulation of RPN1 gene expression by copy number alterations and DNA methylation. **(A)** Likert scale plot showing changes in RPN1 DNA copy number in the pan-cancer dataset from the TCGA database. **(B)** Scatter plot displaying the correlation analysis between RPN1 copy number and RNA expression. **(C)** qPCR detection of the average copy number in several cancer cell lines. Compare with baseline, * P < 0.05, ** P < 0.01, *** P < 0.001. **(D)** Heatmap showing the correlation between RPN1 expression and methylation levels at CpG sites from the TCGA database. * P < 0.05, ** P < 0.01, *** P < 0.001. **(E)** MSP verification of DNA methylation modifications in three cell lines. **(F)** qPCR detection of RPN1 expression changes in three cell lines treated with the DNA methylation inhibitor 5-Aza. Compare with NC, *** P < 0.001. TCGA, The Cancer Genome Atlas. MSP, methylation specific PCR. NS, not significant.

Analysis of pan-cancer DNA copy number data from the TCGA database revealed an increase in RPN1 copy number in the majority of tumor types (**Figure 7A**). Correlation analysis demonstrated a significant positive correlation between RPN1 copy number and RNA expression levels in most tumors (P < 0.05, r > 0.3, **Figure 7B**). The four cancer types with the highest correlation were HNSC (r = 0.675, **Supplementary Figure S9A**), LUSC (r = 0.664, **Supplementary Figure S9B**), ESCA (r = 0.654, **Supplementary Figure S9C**), and LUAD (r = 0.609, **Supplementary Figure S9D**). qPCR analysis confirmed a significant increase in the average RPN1 copy number in several cancer cell lines (**Figure 7C**). On the other hand, based on methylation array data, we identified a significant association between RPN1 expression and methylation levels at specific CpG sites (**Figure 7D**). Using the methPrimer online tool, we designed primers for MSP and identified CpG islands in the promoter region of RPN1 (**Supplementary Figure S9E**). MSP experiments in three cell lines confirmed the presence of DNA methylation modifications in the RPN1 promoter region (**Figure 7E**). As shown in **Figure 7F**, treatment of these cell lines with the DNA methylation inhibitor 5-Aza resulted in increased RPN1 expression, indicating that DNA methylation negatively regulates RPN1 expression.

### 3.8 Regulation of RPN1 expression by transcription factor SP1

**Figure 8:**
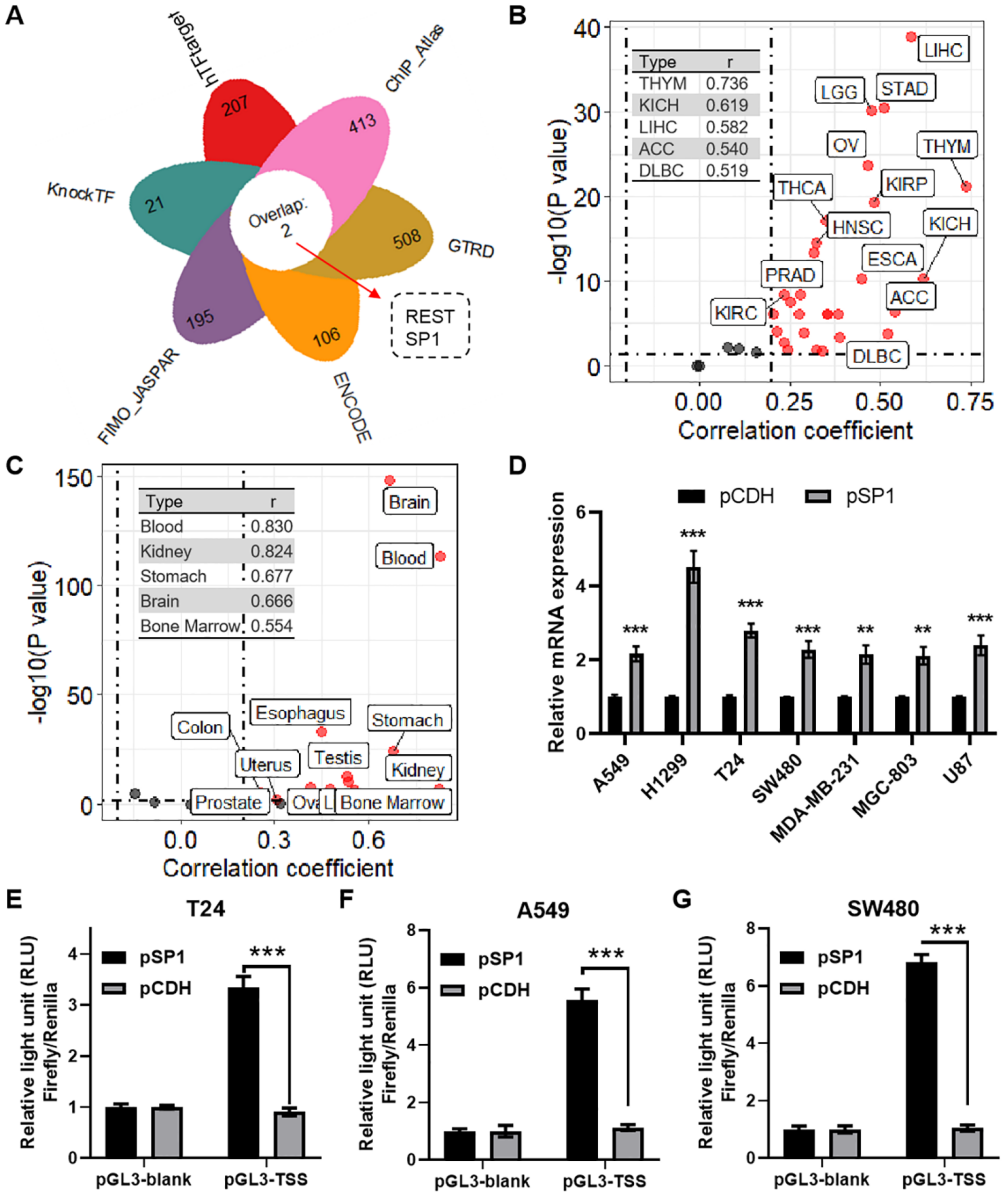
Regulation of RPN1 expression by transcription factor REST. **(A)** Petal chart showing the results of predicting upstream transcription factors of RPN1 using the TFTF package. **(B)** Scatter plot displaying the correlation analysis between RPN1 and SP1 expression in various cancers from the TCGA database. **(C)** Scatter plot showing the correlation analysis between RPN1 and SP1 expression in various tissues from the GTEx database. **(D)** qPCR detection of RPN1 expression changes in cells overexpressing SP1. Dual-luciferase reporter assays validating the transcriptional activation of RPN1 by SP1 in T24 **(E)**, A549 **(F)**, and SW480 **(G)** cells. Compare with pCDH, ** P < 0.01, *** P < 0.001. TCGA, The Cancer Genome Atlas.

Using the TFTF package, we identified SP1 as an upstream transcription factor of RPN1 (**Figure 8A**). Correlation analysis based on pan-cancer data from the TCGA database revealed a positive correlation between the expression of RPN1 and SP1 in the majority of cancers (P < 0.05, r > 0.3, **Figure 8B**). Among them, the cancer types with the highest correlations were THYM (r = 0.736), KICH (r = 0.619), and LIHC (r = 0.582). Similarly, data from the GTEx database demonstrated a positive correlation between RPN1 and SP1 expression across various tissues (P < 0.05, r > 0.3, **Figure 8C**). The tissues with the top three correlation coefficients were blood (r = 0.830), kidney (r = 0.824), and stomach (r = 0.677). qPCR analysis indicated that overexpression of SP1 increased RPN1 expression (**Figure 8D**). Dual-luciferase reporter assays confirmed the transcriptional activation of RPN1 by SP1 in T24 (**Figure 8E**), A549 (**Figure 8F**), and SW480 cells (**Figure 8G**), validating the regulatory role of SP1 on RPN1 transcription.

### 3.9 RPN1 is associated with immunotherapy response

**Figure 9:**
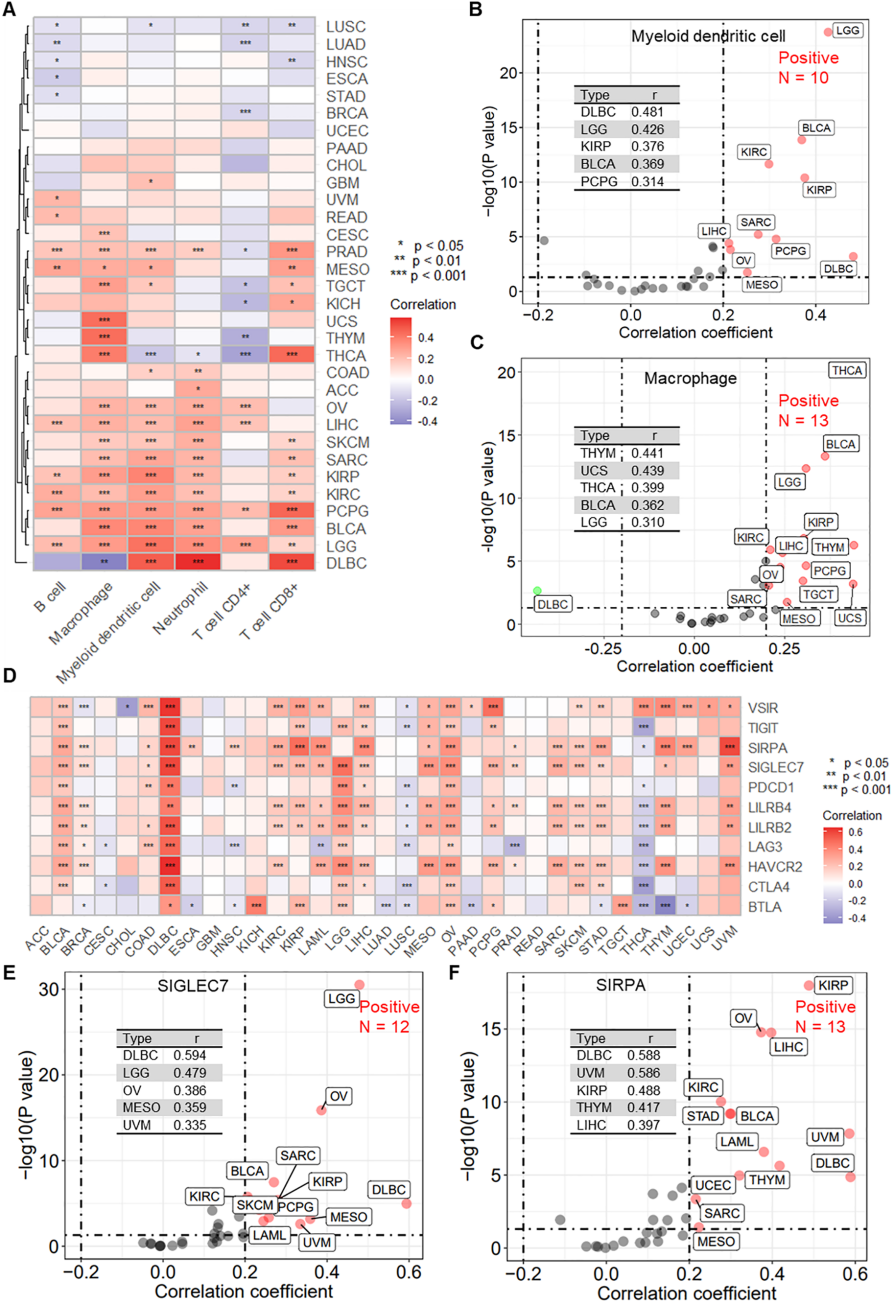
RPN1 is significantly associated with immune cell infiltration and immune checkpoint gene expression. **(A)** Heatmap showing the correlation between RPN1 expression and immune cell infiltration scores obtained using the TIMER algorithm in multiple cancers from the TCGA database. scatter plots showing the correlation analysis results between RPN1 expression and the infiltration scores of myeloid dendritic cells **(B)** and macrophages **(C)**. **(D)** Heatmap showing the correlation between RPN1 expression and 11 immune checkpoint genes in multiple cancers from the TCGA database. Scatter plots showing the correlation analysis results between RPN1 expression and SIGLEC7 **(E)** and SIRPA **(F)**. * P < 0.05,** P < 0.01,*** P < 0.001. TCGA, The Cancer Genome Atlas. TIMER, Tumor Immune Estimation Resource.

Immune therapy response is typically associated with specific immune cell infiltration. Using the TIMER algorithm, we obtained infiltration scores for six major immune cells across pan-cancer samples. Further correlation analysis revealed that RPN1 is significantly associated with the infiltration of various immune cells in multiple cancers (**Figure 9A**). Specifically, for myeloid dendritic cells, high correlation with RPN1 expression was observed in 10 cancer types (r > 0.2, P < 0.05, **Figure 9B**). For macrophages, a high correlation with RPN1 expression was noted in 13 cancer types (r > 0.2, P < 0.05, **Figure 9C**). Additionally, the correlation analysis of immune checkpoint genes (ICGs) expression indicated that RPN1 is correlated with several ICGs across various cancers (**Figure 9D**). For SIGLEC7, 12 cancer types exhibited significant correlation with RPN1 expression (r > 0.2, P < 0.05, **Figure 9E**). Similarly, for SIRPA, a significant correlation with RPN1 expression was found in 12 cancer types (r > 0.2, P < 0.05, **Figure 9F**). Furthermore, we used the TIDE algorithm to assess the immunotherapy sensitivity of tumor samples. Correlation analysis showed that RPN1 expression was significantly positively correlated with the infiltration of tumor-associated fibroblasts (**Supplementary Figure S10A**) and myeloid-derived suppressor cells (**Supplementary Figure S10B**) in multiple cancers. Moreover, RPN1 was significantly negatively correlated with T cell dysfunction (**Supplementary Figure S10C**) and significantly positively correlated with T cell exclusion (**Supplementary Figure S10D**) across various cancers.

## 4 Discussion

Cancer is a highly heterogeneous disease characterized by diverse genetic and molecular features, resulting in distinct malignant phenotypes across different tumor types. Understanding common vulnerabilities and universal characteristics of cancer is crucial for developing more effective and broadly applicable therapeutic strategies. In this context, a pan-cancer perspective has emerged as a powerful approach to identifying key molecular regulators with broad significance in tumor biology. RPN1 is one such pivotal regulator, a protein involved in disulfidoptosis, a recently described form of programmed cell death. Our comprehensive analysis across various cancer types revealed widespread upregulation of RPN1 and its association with poor patient survival outcomes, suggesting a critical role for this protein in driving malignant transformation and disease progression. In this study, we systematically investigated the pan-cancer significance of RPN1, elucidating its functional relevance, transcriptional and epigenetic regulation, and potential as a therapeutic target.

RPN1 is a major component of the oligosaccharyltransferase (OST) complex and is essential for N-linked glycosylation. Previous reports have indicated that alterations in N-glycosylation are crucial for tumorigenesis, proliferation, and metastasis through modification of key proteins or triggering mechanisms involved in maintaining cellular homeostasis (25, 26). Although prior studies have highlighted the role of RPN2 in various cancers (27–29), the impact of RPN1 has been relatively understudied. Notably, since the discovery of disulfidoptosis, RPN1 has garnered increasing attention due to its potential regulatory role in this process. Recent studies have reported the involvement of RPN1 in pan-cancer (30), breast cancer (8, 31), and glioma (32); however, these studies do not provide a comprehensive analysis of the clinical and biological significance of RPN1 and its potential regulatory mechanisms across different cancers. In this study, we confirmed the upregulation of RPN1 in nearly all cancers based on transcriptomic data from the TCGA and GEO databases and proteomic data from the CPTAC database. For patient prognosis, RPN1 was identified as a risk factor for OS, DSS, DFI, and PFI in various tumors.

Chronic ER stress has emerged as a new hallmark of cancer, enabling malignant cells to adapt to oncogenic and environmental challenges while coordinating various immune regulatory mechanisms that promote malignant progression (33). In mammalian cells, three ER membrane proteins serve as sensors of ER stress: Activating Transcription Factor 6 (ATF6), Inositol-Requiring Enzyme 1α (IRE1α, also known as ERN1), and PKR-like ER Kinase (PERK, also known as EIF2AK3) (34). In this study, based on transcriptomic data from the TCGA database and proteomic data from the CPTAC database, we found that RPN1 is associated with ER stress. Dysregulation of the ER stress response leads to an imbalance in protein homeostasis, and increasing evidence links protein homeostasis disruption to cellular senescence (35). Cellular senescence is a permanent state of cell cycle arrest that occurs in proliferating cells under various stresses. Notably, cellular senescence, specifically the presence of senescent cells in the tumor microenvironment, has been classified as a hallmark of cancer (36). In cancer cells, senescence serves as an effective barrier to tumorigenesis (37). Given that the primary biological function of RPN1 involves glycosylation modification, which has been extensively reported to be associated with cellular senescence and senescence-related processes (38, 39), we explored this relationship through bioinformatic analyses. We found that RPN1 is closely related to the cell cycle, and further *in vitro* and *in vivo* experiments confirmed that knockdown of RPN1 inhibits cell proliferation and promotes cellular senescence, evidenced by upregulation of senescence-associated markers P21 and P16, increased β-galactosidase staining, and the senescence-associated secretory phenotype.

At the DNA level, gene expression regulation pathways include copy number variation, DNA methylation, and transcriptional regulation. To assess potential reasons for the upregulation of RPN1 in most tumors, we utilized multi-omics data from TCGA and *in vitro* experiments. We found that RPN1 upregulation is regulated by copy number increases, decreased methylation, and the transcription factor SP1. Specific protein 1 (SP1) is one of the earliest identified transcription factors and a member of the Sp/Kruppel-like factor (Sp/KLF) family. Previous studies have reported that SP1 can activate or repress the transformation of normal cells to cancer cells, thereby promoting or inhibiting cancer progression (40, 41). SP1 is overexpressed in various cancers, including gastric cancer (42, 43), colorectal cancer (44), and lung cancer (45), and is positively correlated with tumor progression. Our study indicates that RPN1 is a target gene of SP1, participating in SP1’s regulatory network in tumors.

Immunotherapy has revolutionized cancer treatment and revitalized the field of tumor immunology (46). Immune cells are the cellular foundation of immunotherapy; hence, understanding immune infiltration in the TME is key to enhancing immunotherapy response rates and developing novel therapeutic strategies (47). Dendritic cells (DCs) exhibit unique antigen-presenting functions and play critical roles in innate and adaptive immune responses. By cross-presenting tumor-associated antigens to naive T cells, DCs contribute to generating specific T cell-mediated anti-tumor effector responses, controlling tumor growth and dissemination (48). In the cancer context, tumor-associated macrophages (TAMs) within the TME typically promote cancer cell proliferation, immunosuppression, and angiogenesis to support tumor growth and metastasis (49). Our analysis identified a correlation between RPN1 expression and infiltration of myeloid dendritic cells, macrophages, and tumor-associated fibroblasts. Furthermore, immune checkpoint blockade can induce durable responses in various cancer types, expanding the curative potential of cancer therapies (50). RPN1 expression was also found to be associated with several immune checkpoint genes, including SIGLEC7 and SIRPA. Based on the TIDE algorithm, we discovered that RPN1 negatively correlates with T cell dysfunction and positively correlates with T cell exclusion. These findings suggest that RPN1 may play a critical role in regulating tumor immunotherapy and immune evasion mechanisms.

This study systematically elucidates the significant role of RPN1 in a pan-cancer context. We found that RPN1 is universally overexpressed and associated with poor prognosis, playing a crucial role in tumor immune evasion. *In vitro* and *in vivo* experiments demonstrated that RPN1 could inhibit tumor progression and promote tumor cell senescence. Additionally, RPN1 expression is regulated at multiple levels, including gene copy number variation, DNA methylation, and transcription factor SP1 regulation. In summary, this study comprehensively reveals the important biological functions of RPN1 as a key pan-cancer regulator, providing a vital basis for developing potential therapeutic strategies targeting RPN1.

## Data Availability

The original contributions presented in the study are included in the article/**Supplementary Material**. Further inquiries can be directed to the corresponding authors.
