# Supplementary material for: Dissecting the functions and regulatory mechanisms of disulfidoptosis-related RPN1 in pan-cancer: modulation of immune microenvironment and cellular senescence
Source: Front Immunol. 2024 Dec 19;15:1512445. doi: 10.3389/fimmu.2024.1512445 (PMC11693735; doi:10.3389/fimmu.2024.1512445)
Supplement: Supplementary file 1 [file DataSheet1.docx]

Supplementary Material

Dissecting the Functions and Regulatory Mechanisms of Disulfidoptosis-Related RPN1 in Pan-Cancer: Modulation of Immune Microenvironment and Cellular Senescence

# Supplementary Figures


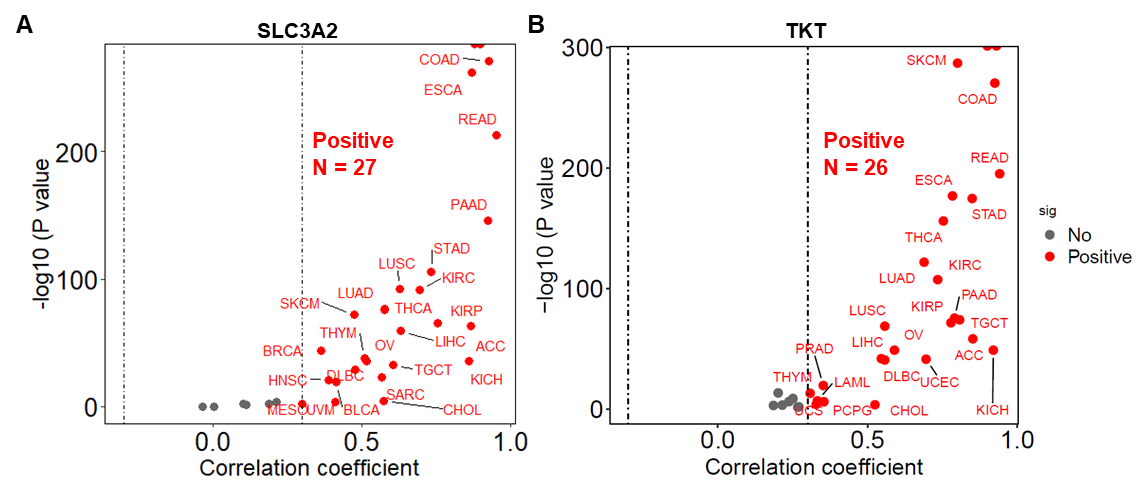


**Figure S1.** Correlation between RPN1 expression and SLC3A2 and TKT expression. Scatter plots show the correlation analysis results of RPN1 expression with SLC3A2 and TKT expression in pan-cancer.


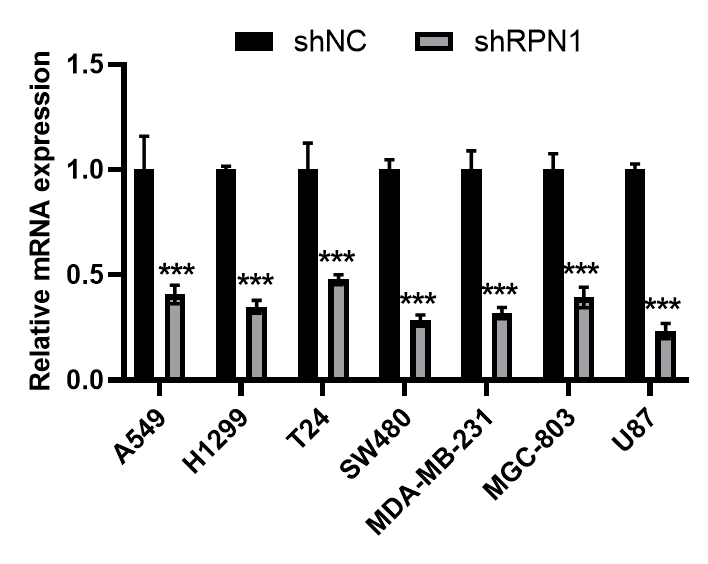


**Figure S2.** qPCR analysis showing changes in RPN1 expression in the seven cell lines following RPN1 knockdown.


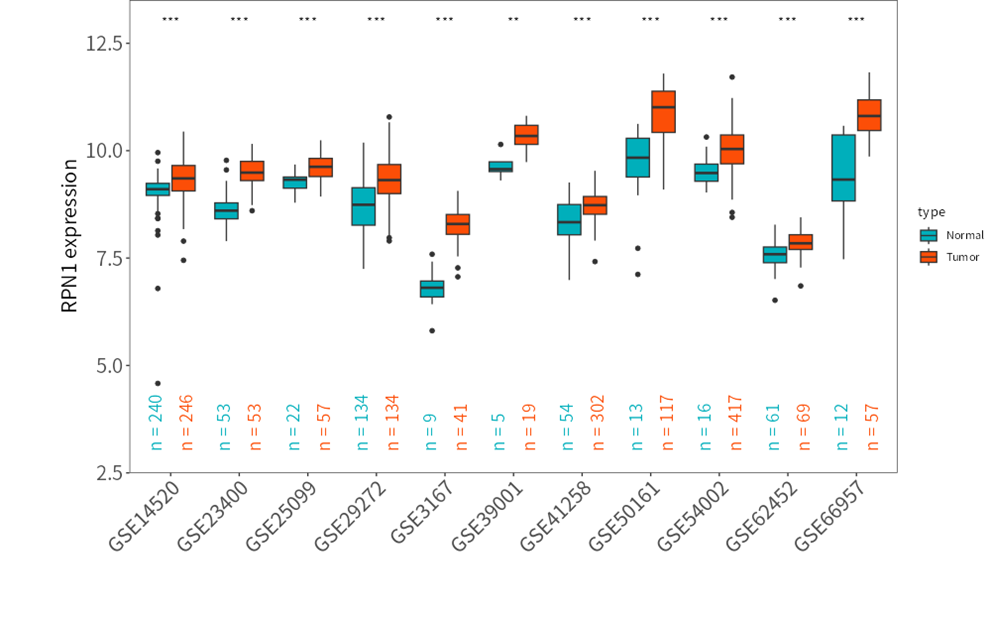


**Figure S3.** Significant upregulation of RPN1 in multiple cancer datasets from the GEO database.


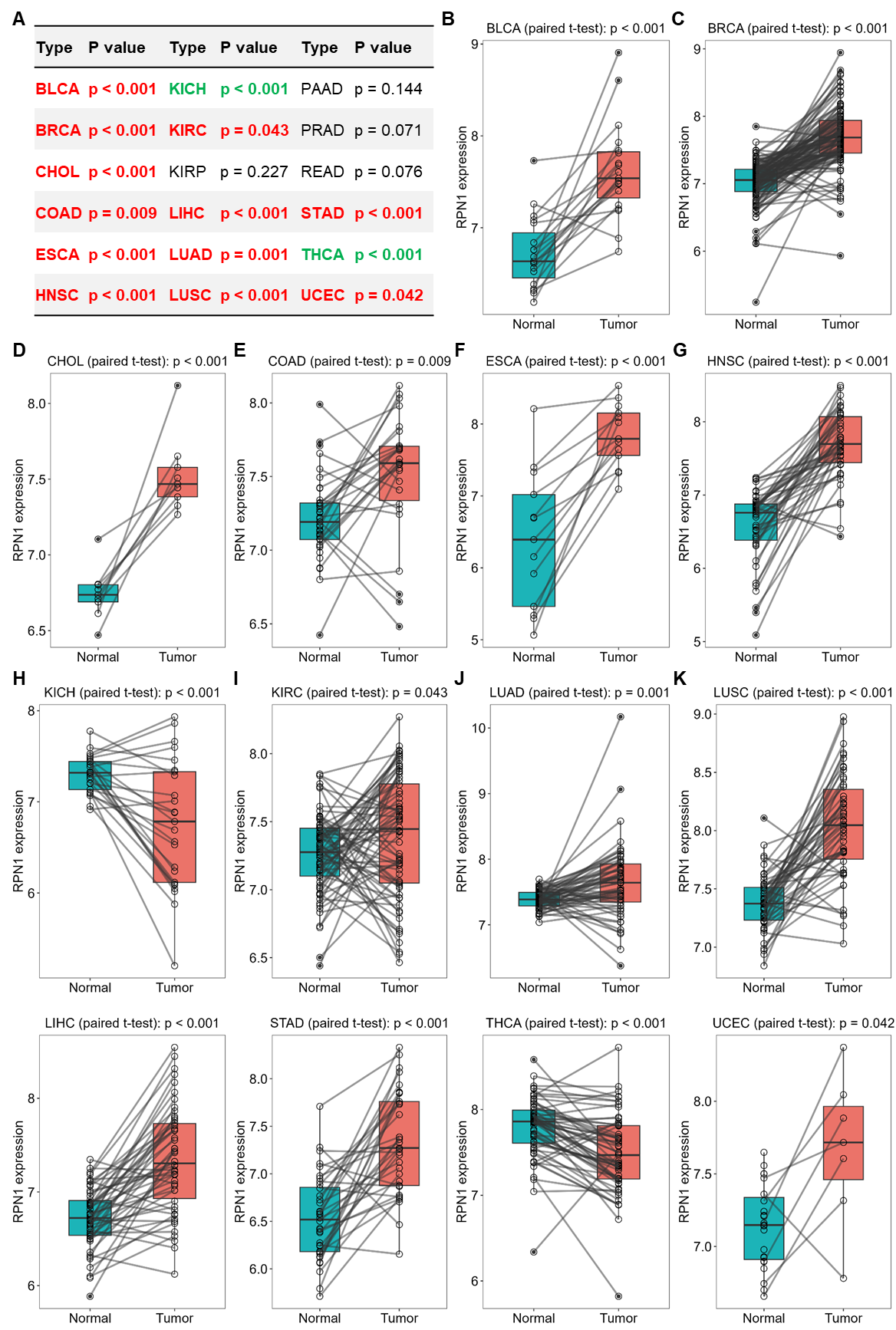


**Figure S4**. RPN1 is upregulated in multiple cancer types based on the results of paired Student’s t-test. (A) Overview of the results of paired Student’s t-test in 18 cancer types. Results of paired Student’s t-test of GAPDH expression in tumor and paired adjacent normal tissues in The Cancer Genome Atlas datasets, including BLCA (B), BRCA (C), CHOL (D), COAD (E), ESCA (F), HNSC (G), KICH (H), KIRC (I), LUAD (J), LUSC (K), LIHC (L), STAD (M), THCA (N) and UCEC (O).


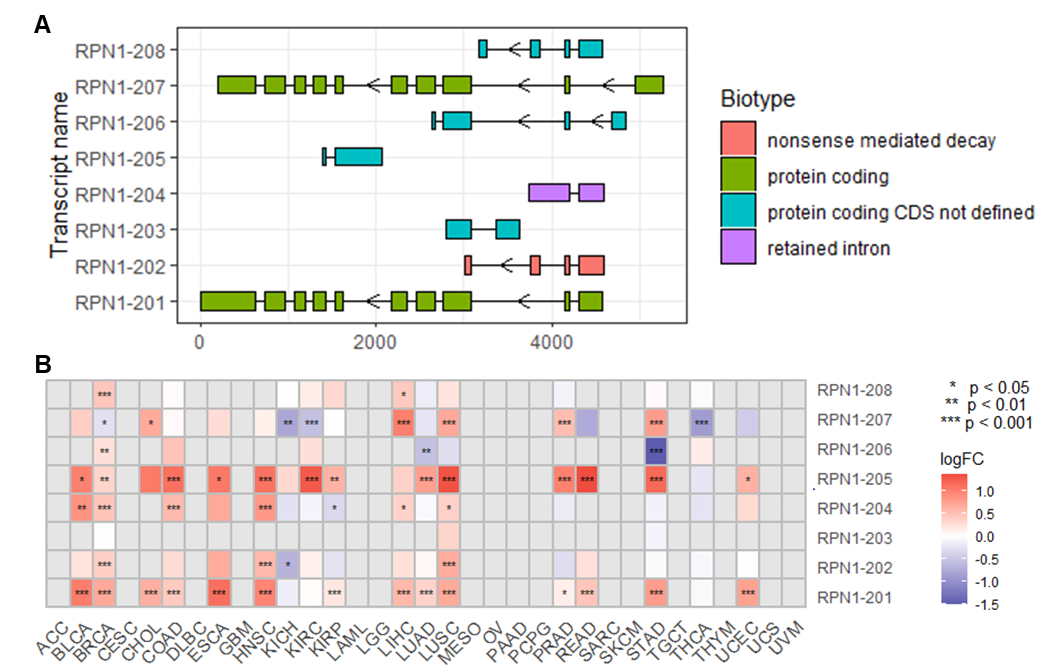


**Figure S5**. Expression of the transcripts in pan-cancer. (A) Overview of the structure of RPN1 transcripts. (B) Heatmap showing the expression change of transcripts of RPN1 in pan-cancer.


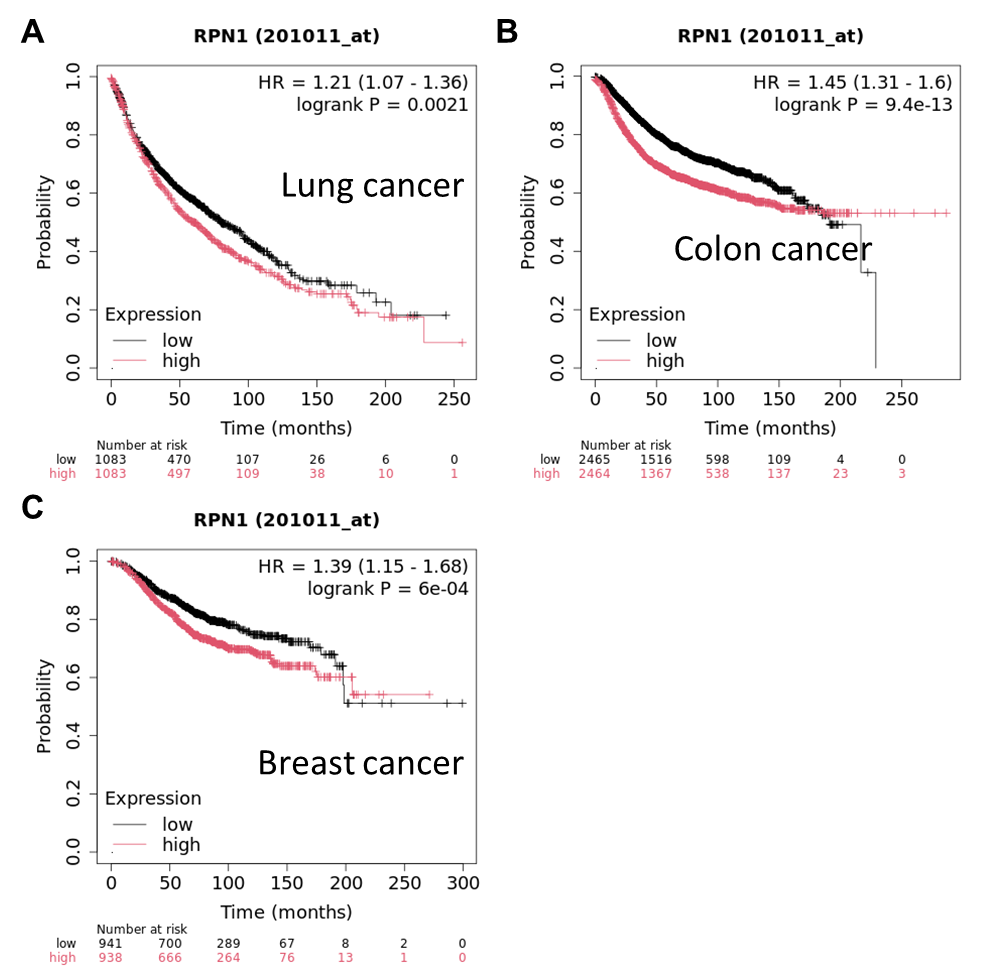


**Figure S6.** RPN1 is a prognostic risk factor in various cancers. Analysis of the impact of RPN1 on overall survival in lung cancer (A), colon cancer (B), and breast cancer (C) using the KMPlotter online tool.


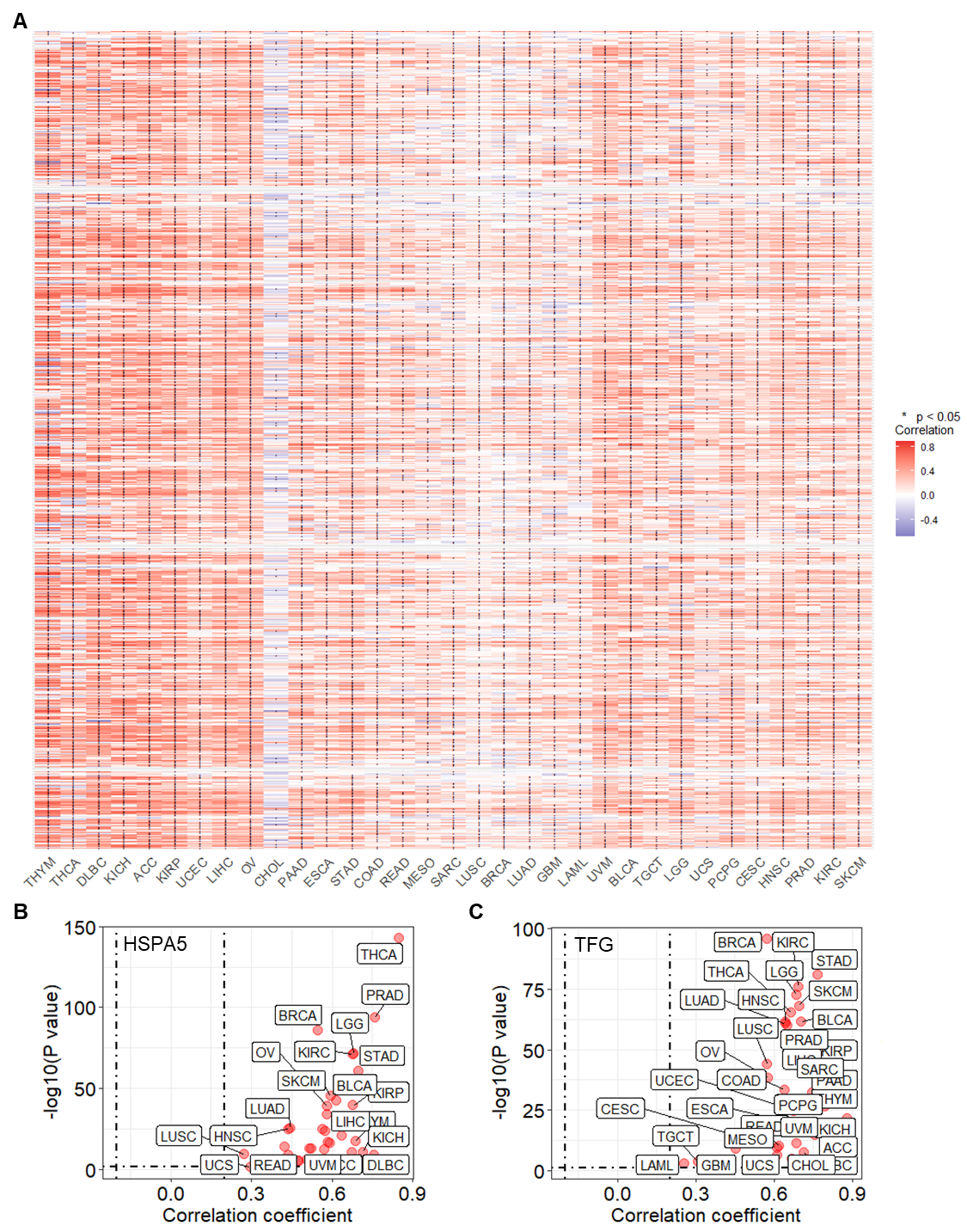


**Figure S7.** Correlation between RPN1 and oncogene expression in pan-cancer. (A) Heatmap showing the correlation analysis results between RPN1 expression and oncogene expression in pan-cancer. Scatter plots show the correlation analysis results of RPN1 expression with HSPA5 (B) and TFG (C) expression in pan-cancer.


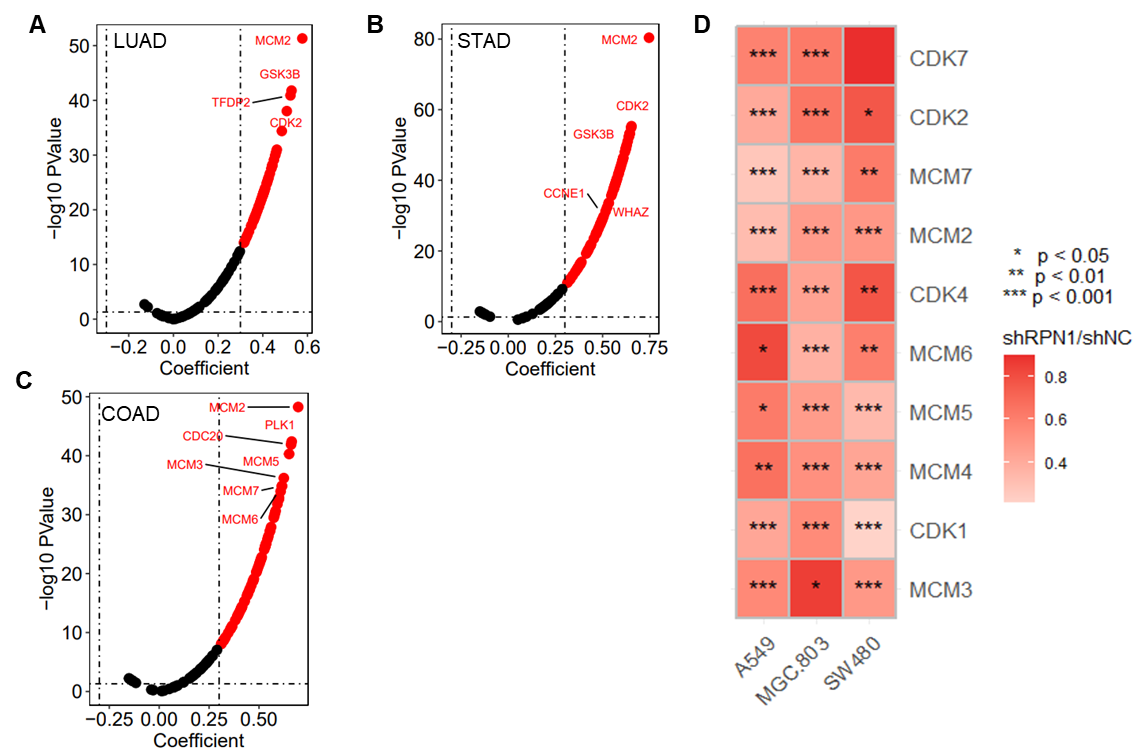


**Figure S8.** Correlation between RPN1 and cell cycle-related gene expression. Scatter plots show the correlation analysis results of RPN1 with cell cycle-related gene expression in TCGA LUAD (A), STAD (B), and COAD (C) datasets. TCGA: The Cancer Genome Atlas; LUAD: lung adenocarcinoma; STAD: stomach adenocarcinoma; COAD: colon adenocarcinoma.


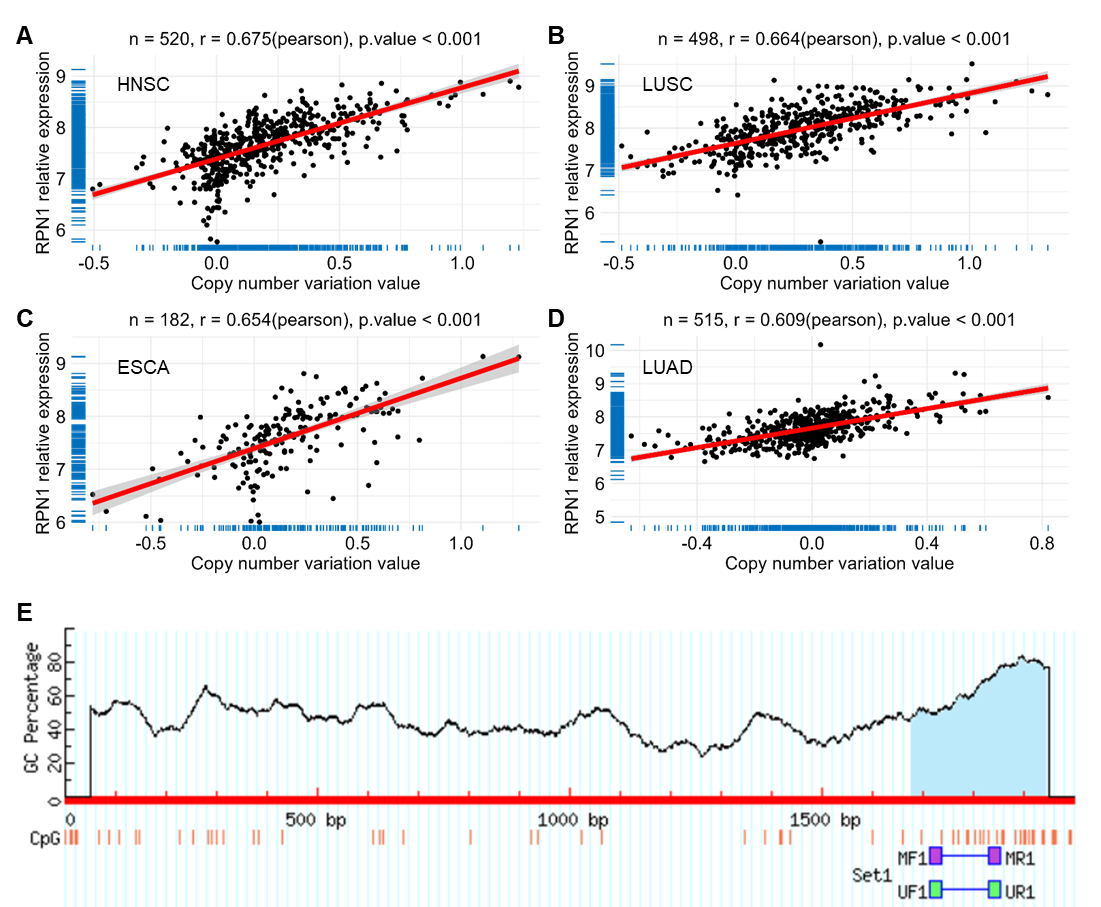


**Figure S9.** Regulation of RPN1 by copy number variation and DNA methylation. Scatter plots show the correlation analysis results of RPN1 expression with gene copy number in HNSC (A), LUSC (B), ESCA (C), and LUAD (D). (E) Prediction of CpG sites in the promoter region of RPN1 and design of methylation-specific PCR primers using the MethPrimer online tool.


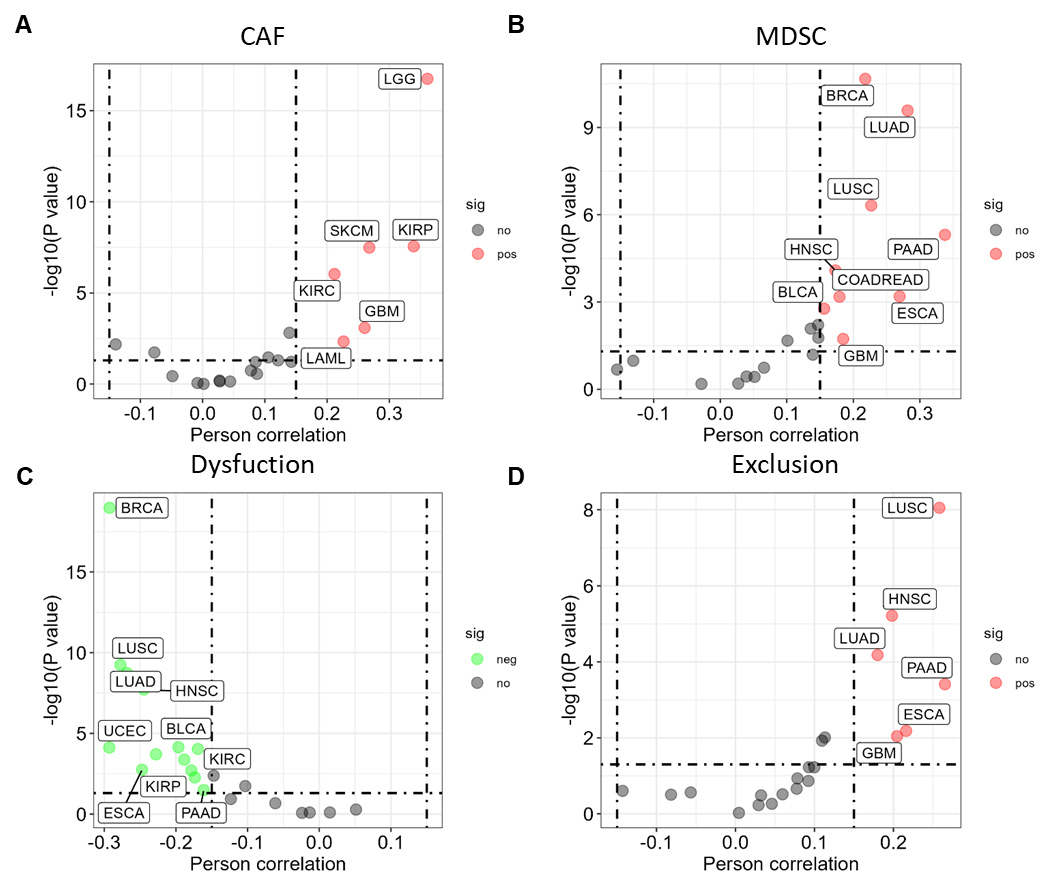


**Figure S10.** Correlation between RPN1 and response to immunotherapy. Scatter plots show the correlation analysis of RPN1 expression with tumor-associated fibroblasts (A), MDSCs (B), T cell dysfunction (C), and T cell exclusion (D) scores. MDSCs: myeloid-derived suppressor cells; CAF: cancer-associated fibroblasts.
